# Epidemiological characteristics of paediatric Omicron infection during the outbreak of SARS-CoV-2 infection during March–May in 2022 in Shanghai, China

**DOI:** 10.1017/S0950268823000663

**Published:** 2023-05-05

**Authors:** Hailing Chang, Xuanzhao Zhang, Hualin Su, Jiehao Cai, Xiaohua Liu, Jingjing Li, Yating Wang, Zhaowen Zhang, Minghui Zhu, Lifang Zhao, Sijia Zhang, Kewen Mei, Liping Zhang, Xiaoguang Wang, Hongjin Yan, Lingfei Luo, Zhiyin Xu, Mei Zeng

**Affiliations:** 1 Department of Infectious Diseases, National Children’s Medical Center, Children’s Hospital of Fudan University, Shanghai, China; 2 Department of Infectious Diseases Control and Prevention, Minhang District Center for Disease Control and Prevention, Shanghai, China; 3 Department of Epidemiology, Key Laboratory of Public Health Safety of Ministry of Education, School of Public Health, Fudan University, Shanghai, China; 4 Department of Planned Immunization, Minhang District Center for Disease Control and Prevention, Shanghai, China; 5 Department of Laboratory Testing, Minhang District Center for Disease Control and Prevention, Shanghai, China; 6 School of Public Health, Key Laboratory of Public Health Safety, Ministry of Education, Fudan University, Shanghai, China

**Keywords:** Children, omicron variant, SARS-CoV-2, epidemiological characteristics, Shanghai

## Abstract

This study aims to understand the epidemiological characteristics of SARS-CoV-2 infection in the paediatric population during the outbreak of the Omicron variant in Shanghai. We retrospectively analysed the population-based epidemiological characteristics and clinical outcome of SARS-CoV-2 Omicron variant infection in children in Minhang District, Shanghai, based on the citywide surveillance system during the outbreak period in 2022 (March to May). During this time, a total of 63,969 cases of SARS-CoV-2 infection were notified in Minhang District, out of which 4,652 (7.3%) were children and adolescents <18 years. The incidence rate of SARS-CoV-2 infections in children was 153 per 10,000. Of all paediatric cases, 50% reported to be clinically symptomatic within 1–3 days after PCR confirmation by parents or themselves, with 36.3% and 18.9% of paediatric cases reporting fever and cough. Also, 58.4% of paediatric cases had received at least one dose of the COVID-19 vaccine and 52.1% had received two doses of the COVID-19 vaccination. Our findings are informative for the implementation of appropriate measures to protect children from the threat of SARS-CoV-2 infection.

## Introduction

The COVID-19 pandemic caused by SARS-CoV-2 has resulted in a global public health crisis and more than 6.6 million deaths [1]. The evolving SARS-CoV-2 variants have caused persistent waves of COVID-19 epidemics worldwide. The SARS-CoV-2 Omicron variant was first identified in South Africa in December 2021 and fuelled the fifth wave of COVID-19 outbreaks across all continents. Compared to previous variants, the Omicron variant causes milder clinical symptoms but is more infectious and transmissible insidiously [2, 3].

In China, inactivated COVID-19 vaccines BBIBP-CorV (Sinopharm) and CoronaVac (Sinovac) were recommended for use in children aged between 3 and <18 years in June 2021. In July 2021, a COVID-19 vaccination program was initiated for the paediatric population across China. The recommended schedule for the primary vaccine series was two doses with an interval of 3–4 weeks. Despite the implementation of strict non-pharmaceutical interventions (NPI), symptom-based active monitoring, contact quarantine, and mass vaccination in Shanghai, the importation of the Omicron variant BA.2 led to the first large outbreak of COVID-19 in Shanghai between March and May 2022 [4]. As of 31 May 2022, approximately 626,000 cases of COVID-19 were reported [5].

During the Omicron wave, a rapid surge of paediatric cases of COVID-19 was observed in the United States, Hong Kong Special Administration Region, and many other parts of the world [6, 7]. Minhang District is the second largest district among the 16 districts of Shanghai and has 10% of the total municipal population. Here, the total number of reported COVID-19 cases were 63,969 from 1 March to 31 May, accounting for around 10% of all reported cases in Shanghai. To understand the epidemiological features of the Omicron variant infection in the paediatric population in Shanghai, we retrospectively analysed all confirmed paediatric cases of COVID-19 reported to the Centers for Disease Control and Prevention (CDC) of Minhang District.

## Methods

A confirmed case is defined as a person who has been diagnosed with PCR-confirmed SARS-CoV-2 infection, regardless of clinical signs and symptoms based on both national and World Health Organization (WHO) guidance [8, 9]. In March 2022, routine PCR screening for SARS-CoV-2 infection was implemented among febrile cases, suspected cases, and close contacts. From 1 April to 31 May, when the Omicron outbreak persisted, universal frequent PCR testing was implemented among all individuals residing in Shanghai. All confirmed cases were mandatorily reported to CDC via the National Infectious Disease Direct Reporting System (NIDDRS) and isolated in hospitals or community isolation facilities.

Once confirmed cases were reported to CDC, a case-by-case field epidemiological investigation was conducted to obtain relevant information, including demographics, clinical symptoms on diagnosis, date of symptom onset, date of the first positive sampling, date of PCR confirmation, date of suspicious contact/exposure, close contacts, and COVID-19 vaccination record. We retrospectively extracted the above-mentioned information from NIDDRS and obtained the COVID-19 vaccination records from the Shanghai COVID-19 Vaccine Immunization Information System.

## Ethics approval

Data collection and analysis were a part of the public health outbreak investigation. Informed consent was exempt from all investigated individuals. The study was approved by the institutional ethical review board.

## Statistical analysis

The incidence rate is defined as the proportion of reported COVID-19 cases in the general population residing in Minhang District. Imported paediatric cases from outside Shanghai were excluded from this study. The demographics of Minhang District population were estimated according to the 2020 Minhang Census data released by the Shanghai municipal government, in which, the total population residing in Minhang District was estimated to be 2,653,489 [10].

Excel 2019 and Stata 16.0 were used to establish a database and for statistical analysis, respectively. Odds ratios (ORs) and 95% confidence intervals (CIs) were calculated using logistic regression analysis to compare the differences in rates between groups. Two-tail *P* values less than 0.05 were considered to be statistically significant.

## Results

From 1 March to 31 May 2022, a total of 63,969 cases of SARS-CoV-2 infection were confirmed in Minhang District, out of which 4,652 (7.3%) cases were of children <18 years old. The incidence rate of children and adolescents <18 years old was 153 per 10,000 (4,652/304,417), which is 0.39 times lower than children compared with that of adults (252 per 10,000, 59,217/2,349,072) (*P* < 0.01). During the Omicron epidemic, 142 (0.22%) severe cases were reported in Minhang District, and 71 (0.11%) cases progressed to death. Fortunately, neither severe nor fatal cases were reported among children and adolescents.

The incidence rate of SARS-CoV-2 infection increased with age among children (Table 1). Children aged 5–10 years account for the largest proportion of children under the age of 18 years, and they also account for the highest number of all paediatric COVID-19 patients (31.5%). There was a significantly higher incidence rate of COVID-19 in children aged 15- < 18 years than in children younger than 5 years (OR: 1.67; 95%CI: 1.62–1.87).

**Table 1.** Incidence rate of COVID-19 in children by age groups from 1 March to 31 May 2022 in Minhang District, Shanghai, China [n (%)]

Age in years	Population	Cases (n = 4,652, %)	Incidence rate (Per 10,000)	*P* value	Odds ratio	95% CI
0 ~ <5^a^	99,533	1,283 (27.6)	128.9	*—*	1	1
5 ~ <10	109,187	1,465 (31.5)	134.2	0.29	1.04	0.97–1.12
10 ~ 15	79,537	1,109 (23.8)	139.4	0.05	1.08	1.00–1.17
15 ~ <18	37,324	795 (17.1)	213.0	<0.001	1.67	1.52–1.82

a Reference category.

**Figure 1.**
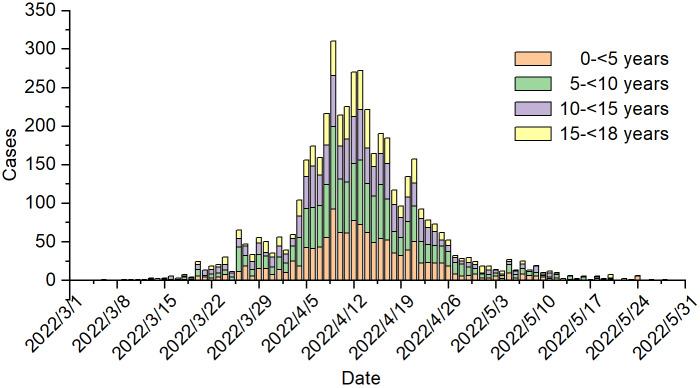
The Daily Number of Children confirmed with SARS-CoV-2 infection in Minhang District from March 1 to May 31, 2022.

The first confirmed paediatric case was reported on 7 March 2022. From 16 March to 31 March, Shanghai implemented a set of targeted interventions and management strategies on a small geographical scale where early cluster outbreaks originated, but it was insufficient to contain the outbreak [11]. As shown in Figure 1, the number of children infected with SARS-CoV-2 in Minhang District started to increase rapidly at the end of March 2022. Although a citywide lockdown was implemented in Shanghai from 1 April, the outbreak peaked among the paediatric population during early-mid April, with 200 to 300 paediatric cases reported daily in Minhang District. Thereafter, the number of paediatric cases began to gradually decrease and occasional cases were reported since the middle of May.

We analysed the clinical manifestation and vaccination status of 4,487 children and adolescents aged <18 years old. The field epidemiological investigation of cases was usually completed within 1 ~ 3 days after PCR confirmation. Thus, the clinical manifestation related to SARS-CoV-2 infection only reflected symptoms within 3 days post infection confirmation. Fifty percent of paediatric cases were reported to be asymptomatic by themselves or by parents, and 50.0% were symptomatic. As shown in Table 2, fever (36.3%), cough (18.9%), runny nose (8.6%), and throat discomfort (5.8%) were the most common clinical symptoms in children. Fever was significantly more commonly reported in young children <5 years than in school-age children 5 ~ <18 years (*χ*^2^ = 104.8, *P* < 0.01). Respiratory symptoms such as cough (χ^2^ = 30.5, *P* < 0.01), throat discomfort (χ^2^ = 93.5, *P* < 0.01), and running nose (χ^2^ = 14.5, *P* < 0.01) were more commonly seen in older children, with a tendency to increase with age. Constitutional symptoms such as myalgia, headaches, and dizziness were also more frequently self-reported in adolescents 12 ~ <18 years than in children <12 years. Regarding vaccination status, 58.4% of paediatric cases had received at least one dose of the COVID-19 vaccine and 52.1% had received two doses of the COVID-19 vaccination. Among children aged 3 ~ <18 years who were eligible for COVID-19 vaccination, 69.7% had received at least one dose of the vaccine. COVID-19 vaccine uptake was significantly lower in preschool children 3 ~ <5 years than in school-age children 5 ~ <18 years (at least one dose of vaccination: 41.8% vs 73.1%, *P* < 0.05; two doses of vaccination: 26.1% vs 72.9%, *P* < 0.05). However, logistic regression analysis with age and sex as control variables did not find a significant association between COVID-19 vaccination and symptomatic infection (OR: 1.0; 95%CI: 0.9–1.2).

**Table 2.** Clinical characteristics and vaccination status of 4,487 children infected with SARS-CoV-2 in Minhang District, Shanghai, China [n (%)]

	0 ~ <5y (n = 1,285)	5 ~ <10y (n = 1,417)	10 ~ <15y (n = 1,039)	15 ~ <18y (n = 746)	Total (n = 4,487)
Sex					
Male	682 (53.1)	754 (53.2)	572 (55.1)	451 (60.5)	2,459 (54.8)
Female	603 (46.9)	663 (46.8)	467 (45.0)	295 (39.5)	2,038 (45.2)
Clinical symptoms^a^					
Any symptoms	695 (54.1)	688 (48.6)	496 (47.7)	363 (48.7)	2,242 (50.0)
Fever	594 (46.2)	505 (35.6)	326 (31.4)	202 (27.1)	1,627 (36.3)
Cough	205 (16.0)	256 (18.1)	220 (21.2)	165 (22.1)	846 (18.9)
Runny nose	83 (6.5)	126 (8.9)	105 (10.1)	72 (9.7)	355 (8.6)
Throat discomfort	28 (2.2)	60 (4.2)	89 (8.6)	85 (11.4)	262 (5.8)
Myalgia	3 (0.2)	11 (0.8)	14 (1.4)	21 (2.8)	49 (1.1)
Fatigue	9 (0.7)	22 (1.6)	11 (1.1)	15 (2.0)	57 (1.3)
Headache or dizziness	5 (0.4)	27 (1.9)	28 (2.7)	41 (5.5)	101 (2.3)
Vomit	14 (1.1)	25 (1.8)	5 (0.5)	1 (0.1)	45 (1.0)
Diarrhea	15 (1.2)	10 (0.7)	4 (0.4)	1 (0.1)	30 (0.7)
Vaccination status					
Unvaccinated	1,052 (81.9)	253 (17.9)	89 (8.6)	164 (22.0)	1,558 (34.7)
1-dose	79 (6.2)	144 (10.2)	22 (2.1)	16 (2.1)	284 (6.3)
2-dose	105 (8.2)	920 (64.9)	862 (83.0)	255 (34.2)	2,338 (52.1)
Missing	49 (3.8)	100 (7.1)	66 (6.4)	311 (41.7)	307 (6.8)

a Clinical symptoms were the relevant symptoms of SARS-CoV-2 infection on epidemiological field investigation, which were self-reported by old children or reported by their parents. For the most confirmed cases, epidemiological field investigation was usually completed within 1–3 days after PCR confirmation.

## Discussion

This study projected a comprehensive epidemiological picture of SARS-CoV-2 infection among the paediatric population in Shanghai during the 2022 Omicron BA.2 wave based on surveillance data from a representative district of Shanghai. Under the implementation of lockdown intervention and partial vaccination, the incidence of SARS-CoV-2 among children and adolescents was 153 per 10,000, which was significantly lower than that reported (19,978/100,000) in the USA during the wave of Omicron outbreak [12]. To some extent, lockdown measures indeed contained the intensity and scale of the Omicron outbreak in Shanghai and protected the paediatric population.

The prevention and control policies and strategies for the COVID-19 pandemic are inconsistent across countries. Facing the persistent challenge of COVID-19 epidemics and the constant emergence of SARS-CoV-2 variants Alpha, Beta, Gamma, and Delta lineage, China adopted a dynamic zero COVID-19 strategy, which enabled the timely identification of SARS-CoV-2 infection cases in the community and control of the outbreak. The evidence showed that the Omicron variant manifests milder clinical symptoms and more asymptomatic infections compared to other variants [2]. Our results are consistent with the current evidence. Based on our investigation data, 50% of paediatric cases were asymptomatic within 1–3 days after PCR confirmation. Either asymptomatic individuals or pre-symptomatic cases played a non-negligible role in the transmission of SARS-CoV-2 [13]. A high proportion of asymptomatic and milder infections of the Omicron variant indeed poses a challenge in trying to understand the impact of NPIs. Earlier studies have illustrated that the majority of COVID-19 incidences may be attributable to silent transmission from a combination of the pre-symptomatic and asymptomatic stages [14]. Another one of our studies showed high levels of household transmission during the Omicron wave in Shanghai due to pre-symptomatic and asymptomatic transmission despite the implementation of strict interventions [15]. This can partly explain why the peak of the Omicron wave among the paediatric and adult population was not interrupted soon after school closure and city lockdown. Hence, the isolation of asymptomatic individuals and quarantine of close contacts are necessary measures to confine the transmission during a large-scale outbreak, which was demonstrated by the modelling study [16].

In the first pandemic wave of COVID-19 in 2020, there was a relatively low attack rate in children and adolescents aged <18 years old, and paediatric cases only accounted for 2.4% of all reported cases in China [17]. Similarly, early surveillance studies from other countries also showed that children only accounted for around 2% of laboratory-confirmed SARS-CoV-2-infected cases once lockdown policies were implemented [18]. However, the incidence of COVID-19 in the paediatric population increased during the persistent pandemic waves over time and with the return to social normalcy. In the United States, a significant rise in paediatric infection was reported in children aged <18 years, representing 17.0%–19.0% of all cases during the Omicron outbreak wave since late December 2021 [19]. As of 27 October 2022, the overall cumulative incidence rate of COVID-19 reached 19,978 cases per 100,000 children in the United States [12]. Given the high transmission of infections of the Omicron variant in school and household settings [15, 20, 21], children and adolescents can be considered to be an important source of infections and play a key role in community transmission. Our recent household transmission study found that infectivity was not significantly different between paediatric primary cases and adult primary cases [15]. Thus, the strict implementation of prevention strategies such as isolation and mask use is required to combat the challenge of the Omicron epidemic in our country at this stage. Also, institutionalised children must receive the COVID-19 vaccination and booster dose as soon as it is possible. Although our data analysis did not find a significant association between COVID-19 vaccination and symptomatic infection, the real-world data on vaccine effectiveness have demonstrated that vaccinating with mRNA vaccines or inactivated vaccines can provide partial protection against Omicron-associated infections and symptomatic infections, and provide relatively high protection against other severe diseases for children [22–25].

Based on the parents’ report and children’s self-report, 50% of Omicron-infected children were reported to have clinical symptoms in the very early period of SARS-CoV-2 infection. The most common clinical symptoms were fever (36.3%) and cough (18.9%). Of note, fever is more commonly seen in children <5 years old compared to children ≥5 years old. In addition, our recent study also showed that getting two doses of the COVID-19 vaccination reduced the risks of symptomatic infection and febrile disease by 35% and 33%, respectively, among children [26]. On 17 June 2022, the Food and Drug Administration (FDA) issued Emergency Use Authorization (EUA) amendments for the mRNA-1273 (Moderna) COVID-19 vaccine for use in children aged 6 months~5 years, and BNT162b2 (Pfizer-BioNTech) COVID-19 vaccine for use in children aged 6 months~4 years [27]. Based on our findings it can be said that ensuring young children aged <3 years receive their COVID-19 vaccination, with the help of expanded immunisation programs, should be a priority because China will inevitably experience large-scale natural infection and outbreak of COVID-19 in the future. During the entire outbreak wave of Omicron in Shanghai, no death was reported among paediatric cases. This may be partly attributable to the high coverage of COVID-19 vaccination among children. In this study, the coverage rate of COVID-19 vaccination is 69.7% in children aged 3 ~ <18 years. The two-dose inactivate COVID-19 vaccine has been demonstrated to be partially effective for symptomatic infection, hospitalisation, and severe disease for a period of 14 days to 5 months since vaccination [23–25]. Facing the challenge of the evolving variation of SARR-CoV-2, the COVID-19 vaccination and the booster vaccination will play a valuable role in protecting the paediatric population against severe disease and hospitalisation.

Upon getting vaccinated, the SARS-CoV-2 Omicron infections among children and adolescents are typically milder or asymptomatic. However, milder symptoms and asymptomatic infection potentially reduce the awareness of individual prevention measures and, consequently, contribute to rapid transmission in the community, household, and school settings. Despite a large outbreak of the Omicron variant in Shanghai, the incidence rate of SARS-CoV-2 infection among the paediatric and the other population was relatively low due to the implementation of strict NPIs. Although enforcing strict NPIs such as school closure protected the majority of the paediatric population from COVID-19 for the time being, this measure interrupted school education, resulting in the accumulation of susceptible children. Once schools and kindergartens re-open after social order returns to normality, the paediatric population will be especially vulnerable to SARS-CoV-2 infection. Therefore, organising massive COVID-19 vaccination camps for children in combination with enforcement of appropriate NPIs will protect the susceptible population in face of epidemics like SARS-CoV-2. Our findings will help inform policy-makers about future prevention strategies for coping with SARS-CoV-2 outbreak in children and adolescents.

This study has a few limitations. First, clinical symptoms of some children and even adolescents were obtained from their parents, and thus, some milder symptoms were possibly under-reported by the parents. Second, the clinical manifestation only reflected symptoms at the time of field investigation within 3 days after infection confirmation, and thus, our data may miss some symptomatic infections developing after investigation. Third, the universal testing for SARS- CoV-2 only began at the end of March 2022, and therefore the real number of infections at the beginning of the COVID-19 outbreak could be underestimated. Above all, we could be underestimating the symptomatic infection of the Omicron variant among paediatric cases. Anyway, ensuring that the paediatric population receives their COVID-19 vaccination should be the top priority in China because the threat of the pandemic still remains.

## Data Availability

All data relevant to the study are available from authors at reasonable request.
